# Antibiotic-Refractory Fever with Pyelonephritis-like Features Revealing Occult Metastatic High-Grade Serous Carcinoma: Competing Causes of Fever and Diagnostic Stewardship

**DOI:** 10.3390/diagnostics16203278

**Published:** 2026-10-09

**Authors:** Wei-Hsuan Huang, Yen-Ling Chang, Hsien-Po Huang, Ting-Kuang Yeh, Chia-Wei Liu, Chien-Hao Tseng, Po-Yu Liu, Po-Hsiu Huang

**Affiliations:** 1 Division of Infectious Diseases, Department of Internal Medicine, Taichung Veterans General Hospital, Taichung 407219, Taiwan; 2 Department of Internal Medicine, Taichung Veterans General Hospital, Taichung 407219, Taiwan; 3 School of Medicine, College of Medicine, National Yang Ming Chiao Tung University, Taipei 112304, Taiwan; 4 Institute of Clinical Medicine, National Yang Ming Chiao Tung University, Taipei 112304, Taiwan; 5 Graduate Institute of Biomedical Engineering, National Chung Hsing University, Taichung 402202, Taiwan; 6 Institute of Molecular Biology, National Chung Hsing University, Taichung 402202, Taiwan; 7 Infection Control Center, Taichung Veterans General Hospital, Taichung 407219, Taiwan; 8 Department of Post-Baccalaureate Medicine, College of Medicine, National Chung Hsing University, Taichung 402202, Taiwan; 9 Doctoral Program in Translational Medicine, Rong Hsing Translational Medicine Research Center, National Chung Hsing University, Taichung 402202, Taiwan

**Keywords:** fever of unknown origin, tumor-associated fever, drug fever, pyuria, pyelonephritis, diagnostic stewardship, antimicrobial stewardship, high-grade serous carcinoma

## Abstract

**Background and Clinical Significance:** Occult malignancy is an easily missed cause of prolonged fever when urinary findings suggest pyelonephritis, and repeated empirical antibiotic courses can delay tissue diagnosis. We report such a case with competing explanations for fever. **Case** **Presentation:** A 62-year-old woman had six months of intermittent fever and one month of dysuria and right flank pain despite nine outpatient antimicrobial courses. On admission, her temperature reached 40.1 °C; she had pyuria with a non-diagnostic urine culture, para-aortic lymphadenopathy on computed tomography and negative blood cultures. Fever abated during intravenous flomoxef, recurred on the seventh day, persisted after its withdrawal and subsided by hospital day 16 before cancer-directed therapy. A single naproxen dose produced only transient defervescence. Nodal biopsy revealed metastatic high-grade serous carcinoma of the tubo-ovarian type (surgical–pathological FIGO stage IIIA1; clinical stage IVB on imaging, not histologically confirmed). The cause of fever remained undetermined; tumor-associated fever, a partially treated urinary infection and flomoxef-associated drug fever were possible contributors. **Conclusions:** When fever persists despite empirical antimicrobials and reassessment finds no convincing bacterial infection, clinicians should consider stopping rather than escalating antibiotics in stable, non-neutropenic patients under close monitoring, consider drug fever and other non-infectious causes, and pursue prompt tissue diagnosis.

## 1. Introduction

Fever of unknown origin (FUO), first systematically characterized by Petersdorf and Beeson [1], remains a demanding diagnostic problem in internal medicine [2,3,4], and malignancy accounts for a minority of cases; a population-based study found that patients hospitalized with FUO have an increased short-term incidence of cancer and that these cancers are somewhat more often disseminated and carry a poorer prognosis [5]. Contemporary studies and reviews continue to identify neoplasia as an important, although numerically smaller, non-infectious cause of prolonged fever [6,7,8,9].

Neoplastic fever, pyrexia attributable directly to a tumor rather than to infection, is a recognized but under-appreciated paraneoplastic phenomenon thought to be mediated by tumor-derived pyrogenic cytokines including interleukin (IL)-1, IL-6, tumor necrosis factor (TNF)-α and interferons, together with pyrogens released during tumor necrosis [10,11]. Because these mediators converge on the same hypothalamic prostaglandin E_2_ pathway as microbial pyrogens, neoplastic and infectious fevers can be clinically indistinguishable [10,12]. Neoplastic fever is therefore a diagnosis of exclusion; the proposed diagnostic criteria require, among other features, the exclusion of infection and allergic drug reactions and a lack of response to at least 7 days of adequate empirical antibiotic therapy [10]. Recent reports continue to describe neoplastic fever as the presenting feature of gynecological and other solid tumors [13,14,15].

To help discriminate the two, Chang and Gross proposed the “naproxen test”, in which a prompt, complete, and sustained normalization of temperature after treatment with naproxen, a non-steroidal anti-inflammatory drug (NSAID), was taken to favor a neoplastic origin [16]; subsequent work has questioned its value, particularly outside populations with already-diagnosed cancer [17]. Diagnosis is further complicated when fever persists despite empirical antimicrobials because the antimicrobials themselves can cause drug fever [18,19,20] and pyuria without bacteriuria has a broad differential diagnosis beyond urinary infection [21,22]. We describe a patient with occult metastatic high-grade serous carcinoma (HGSC) who presented with antibiotic-refractory fever and pyelonephritis-like urinary findings, in whom tumor-associated fever, a partially treated urinary infection and antimicrobial-associated drug fever were competing explanations, and we use the case to discuss the diagnostic management of antibiotic-refractory fever.

## 2. Case Presentation

**Figure 1:**
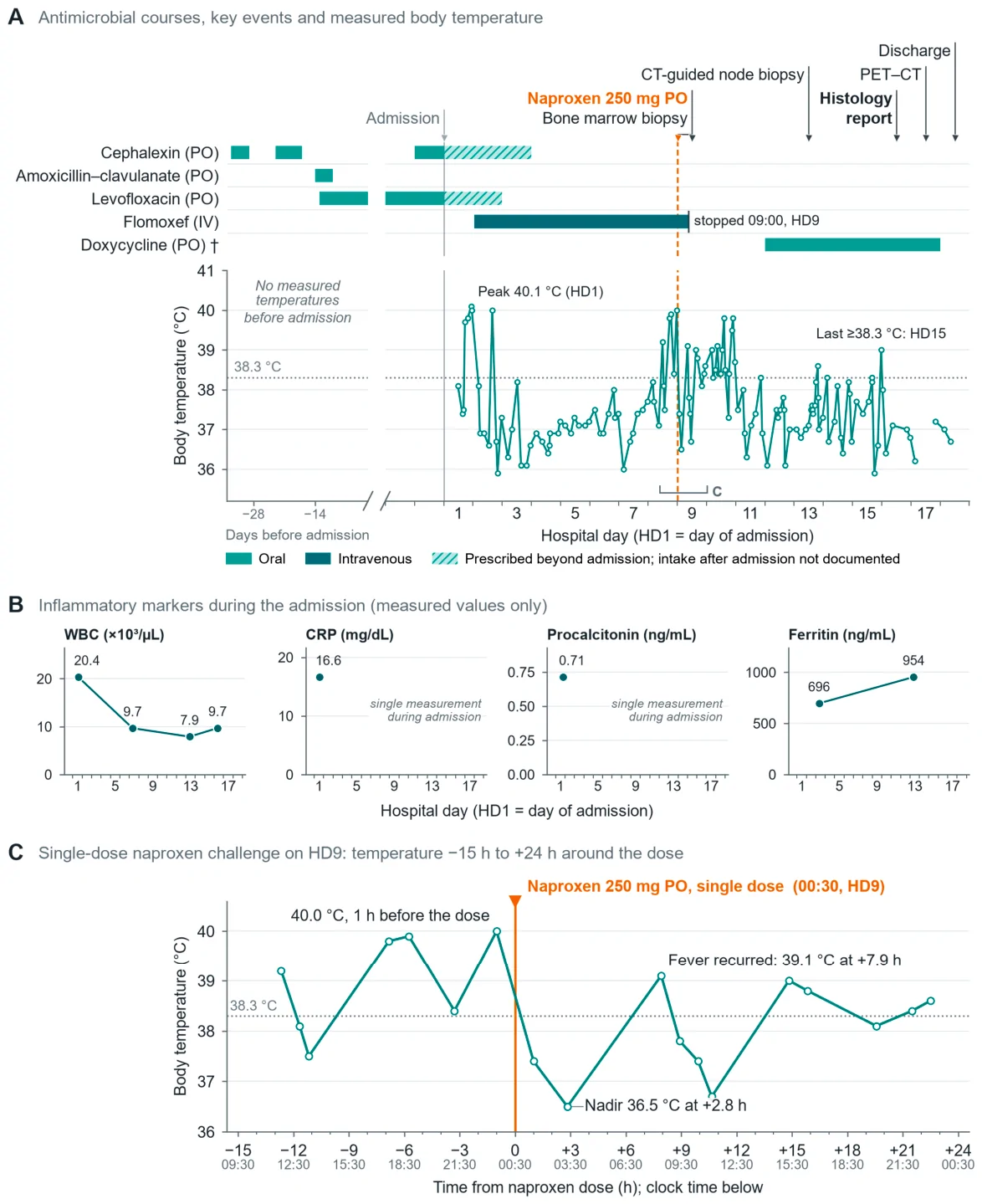
Clinical course during the diagnostic work-up. (**A**) Outpatient antimicrobial courses in the 33 days before admission, inpatient antimicrobials, key diagnostic events and all recorded body temperatures (nursing chart); HD1 is the day of admission. The pre-admission period is compressed (axis break), and no measured temperatures are available for it; four earlier short courses of other oral antimicrobials during the preceding six months are not plotted but are listed in Table 1. Hatched bars indicate outpatient prescriptions extending beyond admission, for which intake after admission was not documented. (**B**) Measured white-cell count (WBC), C-reactive protein (CRP), procalcitonin and ferritin; only values actually measured are shown. (**C**) Body temperature from 15 h before to 24 h after the single 250 mg naproxen dose given at 00:30 on HD9, which occurred before the bone marrow biopsy (HD9) and the nodal biopsy (HD13); flomoxef was discontinued 8.5 h after dose administration. In (**A**,**C**), open circles connected by lines indicate individual recorded temperatures, and the dotted line marks 38.3 °C; in (**B**), filled circles indicate measured values. Events are plotted on their recorded hospital days, with exact times where documented; events without a recorded time are plotted at midday, and courses without recorded times span whole days. † Doxycycline was given for suspected atypical infection. Abbreviations: CT, computed tomography; HD, hospital day; IV, intravenous; PET–CT, positron emission tomography–computed tomography; PO, oral.

A 62-year-old woman presented with approximately six months of intermittent fever and one month of dysuria and right flank pain. Her past history was notable only for laparoscopically assisted vaginal hysterectomy with bilateral salpingo-oophorectomy for uterine myomas and adenomyosis 3 years earlier. During the six months she had received four short courses of oral antimicrobials (erythromycin, amoxicillin, doxycycline and clindamycin) at other clinics, and during the final month, she received five courses for presumed urinary tract infection—three of cephalexin, one of amoxicillin–clavulanate and one of levofloxacin, with the last three overlapping in part—covering 24 of the 33 days before admission, without improvement (Figure 1A; Table 1). According to the patient, the fever had begun before the first of these courses. There was no history of malignancy in first-degree relatives, and her psychosocial history was non-contributory. On admission (hospital day [HD] 1), her triage temperature was 38.1 °C and examination revealed right costovertebral-angle tenderness.

**Figure 2:**
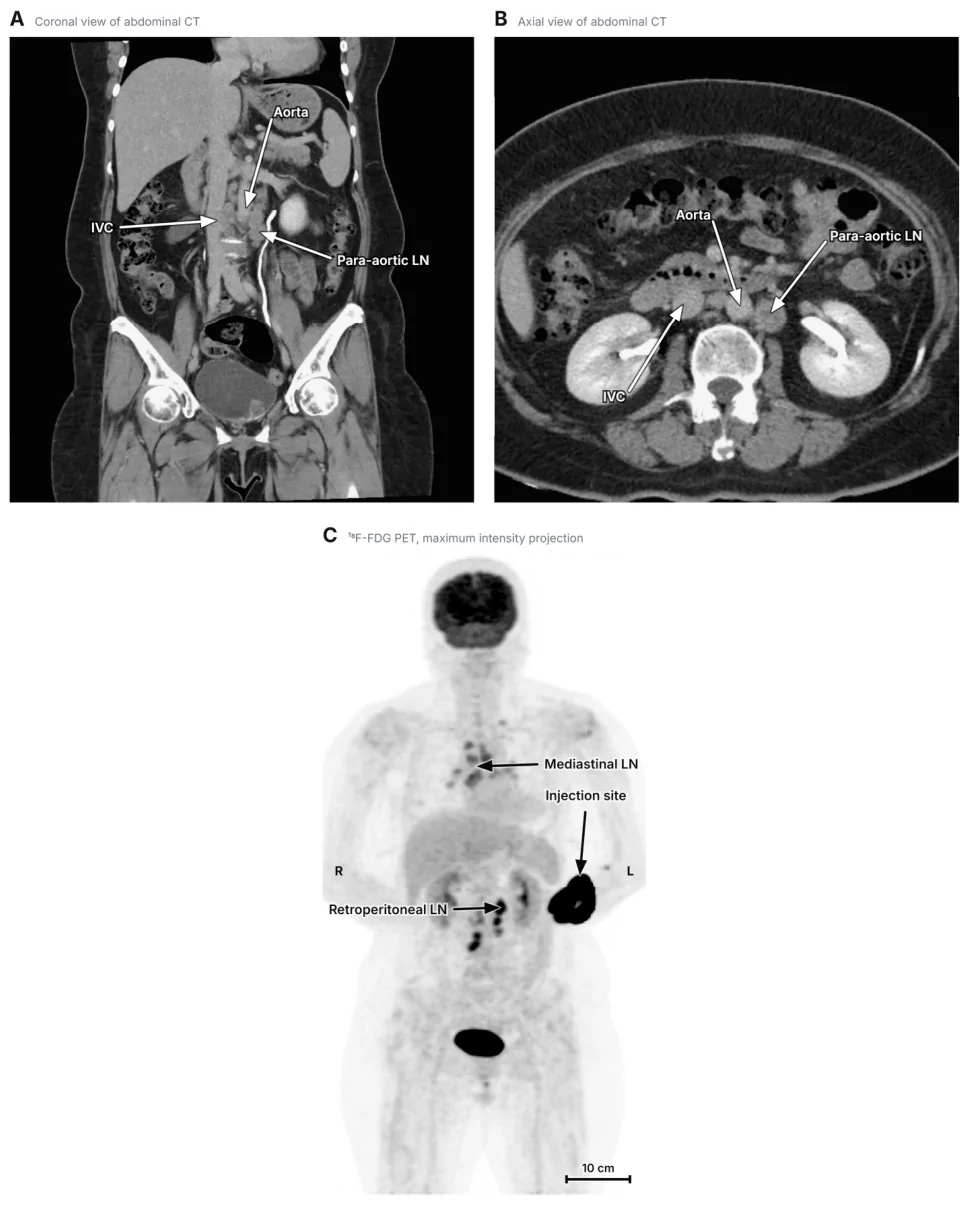
Imaging findings at diagnosis. (**A**) Coronal contrast-enhanced abdominal computed tomography (CT) showing enlarged para-aortic retroperitoneal lymph nodes; the aorta and inferior vena cava (IVC) are labeled for orientation. (**B**) Corresponding axial CT at the level of the kidneys, showing para-aortic nodal tissue adjacent to the aorta. (**C**) Whole-body ^18^F-fluorodeoxyglucose positron emission tomography maximum-intensity projection demonstrating hypermetabolic retroperitoneal nodal disease and mediastinal uptake; together with the left supraclavicular nodal uptake (not annotated), these extra-abdominal findings were suggestive of, but not histologically confirmed as, nodal metastases. Arrows mark representative lesions; not every involved site is annotated. The focus of intense uptake projected over the left upper abdomen corresponds to the left upper-extremity tracer injection site and should not be mistaken for a metastatic deposit. R, right; L, left. The 10 cm scale bar applies to panel (**C**) only; no scale marker is available for panels (**A**,**B**), which should be interpreted using the labeled anatomical landmarks.

Laboratory studies showed leukocytosis (white-cell count: 20,350/µL, 79% neutrophils), a C-reactive protein (CRP) concentration of 16.6 mg/dL and a procalcitonin concentration of 0.71 ng/mL; ferritin was 696 ng/mL on HD3. Urinalysis showed pyuria (20–29 white cells per high-power field; leukocyte esterase 2+), microscopic hematuria (6–9 red cells per high-power field) and a negative nitrite test; the urine culture yielded only Gram-positive bacilli and was considered non-diagnostic, and renal function was normal. Contrast-enhanced abdominal computed tomography (CT) revealed multiple enlarged para-aortic retroperitoneal lymph nodes (Figure 2A,B) without hydronephrosis. Empirical intravenous flomoxef (1 g every 8 h) was begun early on HD2 for presumed refractory pyelonephritis.

Later on HD1, before intravenous therapy, the temperature rose to 40.1 °C, and 40.0 °C was recorded again on HD2. From HD3 to HD7, no temperature ≥38.3 °C was recorded (maximum: 38.2 °C). Fever recurred on HD8, the seventh day of flomoxef treatment, with peaks of 39.2–40.0 °C (Figure 1A). During high fever, the pulse rate was 99–117 beats/min at 39.5–40.0 °C, values lower than expected for the degree of fever according to the conventional pulse–temperature relationship (relative bradycardia), both before and during flomoxef therapy; the patient had chills but did not appear toxic. Blood cultures and sputum culture were negative, the intravenous-catheter site was unremarkable, there was no diarrhea, chest imaging showed no pneumonia, and the D-dimer level was elevated on HD3, although no venous imaging was performed. There was no rash, eosinophils remained at 0–0.8% of leukocytes, hepatic and renal function remained normal, and the white-cell count had fallen to 9660/µL by HD7 (Figure 1B). Transthoracic echocardiography on HD6 showed a thickened aortic valve with mild regurgitation and preserved left-ventricular systolic function; no vegetation was reported. Ferritin levels rose to 954 ng/mL and lactate dehydrogenase levels increased from 190 to 294 U/L by HD13. Antinuclear antibody was positive at 1:320 (fine speckled pattern), and anti-SSA/Ro52 and antimitochondrial antibodies were also positive; because rheumatological assessment found no clinical features of connective-tissue disease, no treatment was given.

At 00:30 on HD9, a single oral 250 mg dose of naproxen was given as an informal trial 1 h after a temperature of 40.0 °C; the bone marrow and nodal biopsies had already been scheduled. The temperature fell to 36.5 °C at 2.8 h and rose again to 39.1 °C at 7.9 h (Figure 1C). The bone marrow biopsy, which was performed later on HD9 to evaluate for a hematologic malignancy, showed a hypercellular marrow (cellularity approximately 60%) with increased but normally maturing trilineage hematopoiesis, blasts accounting for about 1% of nucleated cells, negative in situ hybridization for Epstein–Barr virus-encoded RNA, and no evidence of malignant infiltration. Flomoxef was discontinued at 09:00 on HD9 after 7 days of therapy because no infectious source had been identified and fever had recurred despite treatment; acetaminophen, which was administered as needed, was the only antipyretic given thereafter. High fever (>39.0 °C; up to 39.8 °C) persisted through HD10 and then abated; isolated temperatures of 38.3–39.0 °C were recorded on HD11 and HD13–HD15, including one on the day of the nodal biopsy and none from HD16 onward. Oral doxycycline was given on HD12–HD17 for suspected atypical infection; serological tests for atypical pathogens were negative.

CT-guided biopsy of a para-aortic node on HD13 (Figure 2A,B) yielded metastatic adenocarcinoma, which was reported on HD16. Immunohistochemistry was positive for PAX8, WT1, and the estrogen receptor (weak), with p53 overexpression, only scant GATA3 staining (<5% of tumor cells), and negative staining for TTF-1, consistent with HGSC of the tubo-ovarian type; because bilateral salpingo-oophorectomy had been performed 3 years earlier, the primary site could not be established; most HGSCs are now thought to originate in the fallopian tube [23], and HGSC can present with nodal metastases without a clinically or radiologically detectable primary tumor [24]. Acid-fast staining of the nodal biopsy tissue and three sputum specimens was negative, and mycobacterial cultures of the nodal biopsy, the three sputum specimens and a single urine specimen ultimately showed no growth; no nucleic acid amplification test for tuberculosis or interferon-γ release assay was performed. Serum cancer antigen 125 (CA-125) level was 2265 U/mL. Whole-body ^18^F-fluorodeoxyglucose positron emission tomography–computed tomography (PET–CT) on HD17 showed intense uptake in the retroperitoneal and left supraclavicular nodes (maximum standardized uptake value: 13.9), reported as probably malignant; uptake in mediastinal, bilateral pulmonary hilar and presacral regions, reported as possibly malignant, with inflammation, infection or granulation tissue as alternative explanations; and equivocal uptake in the right supraclavicular and right distal external iliac regions (Figure 2C). She was discharged on HD18.

**Figure 3:**
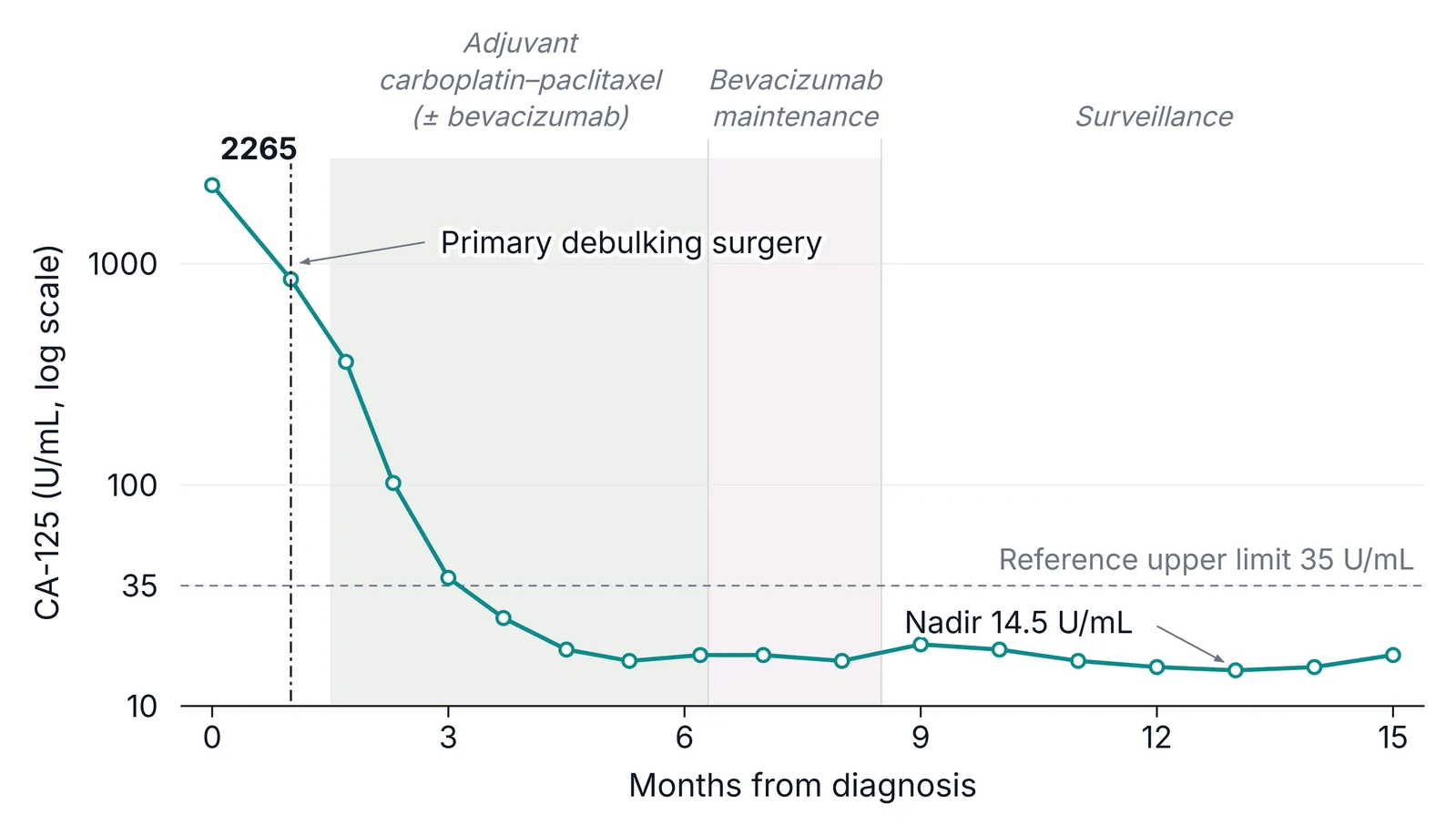
Serum CA-125 levels (log scale) from diagnosis through primary debulking surgery (dash-dotted line), adjuvant carboplatin–paclitaxel with bevacizumab from the third cycle and bevacizumab maintenance (shaded areas), and surveillance. CA-125 levels fell from 2265 U/mL at diagnosis, normalized within about 4 months and thereafter remained below the reference upper limit of 35 U/mL (dashed line) through 15 months of follow-up, with a nadir of 14.5 U/mL. Abbreviations: CA-125, cancer antigen 125.

Twelve days after discharge (day 30, counting the day of admission as day 1), before surgery, a repeat urinalysis showed no pyuria (0–5 white cells per high-power field; leukocyte esterase negative). Primary optimal debulking surgery, including bilateral pelvic and para-aortic lymphadenectomy with omentectomy, was performed on day 41. At surgery there were no ascites and no gross tumor on the peritoneum, omentum or appendix; enlarged para-aortic, intercaval and presacral lymph nodes extended to the level of the renal veins, and no gross residual intra-abdominal disease remained. Metastatic HGSC was confirmed in 14 of 23 para-aortic, intercaval and presacral lymph nodes (right para-aortic: 1/1, left high para-aortic: 6/9, intercaval: 5/9, and presacral: 2/4), whereas the right and left pelvic nodes (common iliac, external iliac, hypogastric and obturator; 0/6 and 0/8) and the omentum were uninvolved (14 of 37 retroperitoneal nodes overall); no granulomatous inflammation was described, and the lymphadenectomy specimens were not submitted for mycobacterial testing. This corresponds to surgical–pathological stage IIIA1 according to the International Federation of Gynecology and Obstetrics (FIGO) 2014 classification for cancer of the ovary, fallopian tube and peritoneum [25], which was applied by convention because the adnexa had been removed previously; the substage was not assigned because the size of the largest nodal metastasis was not recorded. Because preoperative PET–CT had suggested extra-abdominal (left supraclavicular and mediastinal) nodal involvement, the disease was clinically classified as FIGO stage IVB, although these nodes were not histologically confirmed. She was readmitted on day 56 with a postoperative urinary tract infection; after recovery, no fever was documented during chemotherapy or follow-up. Six cycles of adjuvant carboplatin–paclitaxel scheduled every 3 weeks, with bevacizumab added from the third cycle, were completed and followed by bevacizumab maintenance; CA-125 levels fell rapidly, normalized within about 4 months, reached a nadir of 14.5 U/mL and has remained below the reference upper limit of 35 U/mL through 15 months of follow-up (Figure 3). Computed tomography after the third cycle of chemotherapy showed regression of the mediastinal and hilar lymph nodes without antituberculous therapy, and no tuberculosis was diagnosed during follow-up.

Patient perspective. The patient recalled that the most difficult part of her illness was the months of recurrent fever during which repeated courses of antibiotics gave no relief and no cause could be identified. She reported that being given a definite diagnosis after the biopsy, although the news itself was hard to hear, was less distressing than the preceding uncertainty and that the establishment of a definite diagnosis and the absence of recurrent fever during cancer treatment reassured her that the underlying problem was finally being addressed. She agreed to the publication of this report in the hope that other patients with unexplained fever might be investigated for malignancy sooner.

## 3. Discussion

The contribution of malignancy to the pyelonephritis-like presentation remains uncertain. The retroperitoneal nodal disease, which at surgery involved the right para-aortic and intercaval as well as left para-aortic nodes up to the level of the renal veins, may have contributed to the right flank pain and costovertebral-angle tenderness, even in the absence of hydronephrosis, although the nodes illustrated on the admission CT lay to the left of the aorta (Figure 2A,B), so this link is not established. Nodal disease would not, however, readily explain dysuria or pyuria, and pyuria without bacteriuria has a broad differential diagnosis that includes current or recent antimicrobial therapy, genitourinary tuberculosis and inflammatory conditions adjacent to the urinary tract [21], and sterile pyuria is frequent even among hospitalized patients without urinary infection [26]. Pyuria with microscopic hematuria was documented once in the hospital, after outpatient antimicrobial prescriptions covering 24 of the preceding 33 days, and had resolved by day 30, after flomoxef and doxycycline treatment but before any tumor-directed treatment—a course compatible with a partially treated urinary tract infection or with pyuria related to antimicrobial exposure rather than with a tumor-related process; no urine culture obtained before the first course prescribed for the presumed urinary tract infection (day −33) was available. We therefore regard the flank findings as possibly tumor-related and the urinary findings as non-specific: together they were compatible with, but not diagnostic of, pyelonephritis. The practical point is that such findings should not anchor the diagnosis when fever fails to respond to appropriate therapy.

The cause of the fever remained undetermined and may have been multifactorial; the explanations considered are summarized in Table 2. In favor of tumor-associated fever, intermittent fever had been present for about six months and, by the patient’s account, preceded all antimicrobial exposure, persisted through nine outpatient courses of antimicrobials of six classes and reached 40.1 °C on admission before intravenous therapy (although outpatient oral antimicrobials had been taken until admission), and no persistent infectious focus was identified in a patient with metastatic HGSC. Several findings nevertheless limited this attribution. The fever subsided by HD16, before any cancer-directed therapy, about 1 week after flomoxef was withdrawn and on the fifth day of doxycycline; this resolution, together with competing explanations for defervescence, weakened the attribution to the malignancy and precluded its confirmation through a response to tumor-directed treatment. Temperatures were not systematically recorded between discharge and the start of chemotherapy. The evidence that fever preceded all antimicrobials rests solely on the patient’s recall, without measured temperatures, and the proposed diagnostic criteria for neoplastic fever, which require the exclusion of allergic drug reactions and a sustained response to a naproxen test performed according to its protocol [10], were not fulfilled. The diagnosis of metastatic HGSC therefore does not by itself establish that the tumor caused the fever. Relative bradycardia was present both before and during flomoxef therapy and therefore did not discriminate between explanations, and the rise in lactate dehydrogenase was non-specific, as it is a common phenomenon in antibiotic-induced drug fever [19]. Cytokine profiling was not performed, although tumor-derived IL-6 has been implicated in paraneoplastic fever in solid cancers [11].

Two other explanations also remained plausible. A partially treated urinary tract infection may have contributed at presentation: pyuria and leukocytosis were present on admission, and both the fever and the white-cell count fell during the first days of flomoxef treatment (HD3–HD7). This defervescence, however, also coincided with the end of the outpatient levofloxacin and cephalexin prescriptions (intake after admission was not documented; Figure 1A), so it cannot be attributed to treatment with confidence, and drug fever from these oral agents may have also contributed to the fever at presentation. Drug fever from flomoxef therapy may have contributed to the recrudescence that began on HD8, the seventh day of therapy (about 6.5 days after the first dose), which is close to the 7–10-day interval typical in non-sensitized patients [18]; in a series of patients receiving parenteral antibiotics, in which β-lactams were the antibiotics most often implicated, previous β-lactam therapy, which this patient had also received, was associated with a shorter onset interval for piperacillin (median 13 vs. 22.5 days) but not for cefotaxime [19], so this observation cannot be extrapolated directly to flomoxef. The absence of a rash and eosinophilia does not exclude the diagnosis because these features are infrequent [27]. However, fever had been present before flomoxef treatment was started; although high fever abated within about 36 h of withdrawal, as would be expected for drug fever, intermittent temperatures ≥38.3 °C continued until HD15, about 6.5 days after withdrawal, which is longer than the rapid resolution characteristic of drug fever [18,19,20]; and, in the same series, peaks of 40 °C or higher were rare and drug fever was less frequent in patients with malignancy [19]. For the recrudescence observed during flomoxef therapy, the Naranjo score [28] was four, indicating a “possible” adverse drug reaction (previous reports of β-lactam drug fever [19,29], +1; onset after the drug was started, +2; partial improvement after withdrawal, +1; alternative causes present, −1; objectively documented fever, +1; no rechallenge). We therefore regard tumor-associated fever, a partially treated urinary tract infection at presentation and flomoxef-associated drug fever during the recrudescence as possible, not mutually exclusive, explanations, none of which could be confirmed.

Other explanations were considered less likely (Table 2). Culture-negative endocarditis was not fully excluded because only transthoracic echocardiography was performed; an atypical or rickettsial infection was not supported by serology, and febrile readings continued on days 2–4 of doxycycline treatment (HD13–HD15) before the fever subsided on its fifth day; and venous thromboembolism was not excluded because the D-dimer level was elevated and no venous imaging was performed. The positive autoantibodies, without clinical features of connective-tissue disease, were of uncertain significance.

Tuberculosis required particular consideration in this endemic setting. Pyuria without bacteriuria is a recognized presentation of genitourinary tuberculosis [21], and the microscopic hematuria, prolonged fever, lymphadenopathy and FDG-avid mediastinal and hilar nodes were also compatible with tuberculosis. The preceding treatment with levofloxacin, which is active against *Mycobacterium* *tuberculosis*, was considered a potential confounder when interpreting the clinical course, although whether it affected an unrecognized mycobacterial infection in this patient could not be determined. Against tuberculosis, acid-fast staining of the nodal biopsy and three sputum specimens was negative, and mycobacterial cultures of the nodal biopsy, the three sputum specimens and a single urine specimen showed no growth; no granulomatous inflammation was described in the surgical pathology report of the 37 resected retroperitoneal nodes; and the mediastinal and hilar nodes regressed after three cycles of chemotherapy without antituberculous therapy, with no tuberculosis diagnosed during 15 months of follow-up. The evaluation was nevertheless incomplete, as listed among the limitations, and we therefore consider tuberculosis unlikely but not excluded.

The single-dose naproxen challenge should be interpreted cautiously (Table 3). It was given as an informal trial before both biopsies, which had already been scheduled, and did not influence the decision to obtain tissue. The original naproxen test was derived mainly in patients with known cancer (20 of 22) and used continued dosing (250 mg twice daily), with “sustained” defervescence defined as remaining afebrile for more than 5 days while naproxen was continued [10,16]; Chang’s later protocol specifies 375 mg every 12 h for at least 36 h after at least 7 days of adequate antibiotic therapy [10]. A single dose in a patient without a cancer diagnosis who was receiving a β-lactam that may itself cause fever—and whose flomoxef treatment was stopped 8.5 h after the dose—corresponds to neither the protocol nor the derivation population, so the transient response is interpretable as neither positive nor negative. NSAID antipyresis did not discriminate neoplastic from non-neoplastic fever in unselected patients with prolonged fever [17], and pooled data from patients with cancer describe treatment responses rather than diagnostic accuracy [30].

Framed as diagnostic stewardship [31], the main lessons concern fever that does not respond to empirical antimicrobials. Over the six months before admission, the patient had received nine outpatient courses of oral antimicrobials from six classes, including prescriptions covering 24 of the last 33 days. In the hospital she received a further 7 days of flomoxef, during which fever abated only transiently, and 6 days of doxycycline; fever resolved on the fifth day of doxycycline treatment, although whether doxycycline contributed to defervescence remains uncertain. Current guidance recommends short courses (5–7 days of a fluoroquinolone or 7 days of another agent) for complicated urinary tract infection in patients who are improving on effective therapy [32]; this patient did not improve on the outpatient courses, and her lack of response called for diagnostic reassessment rather than further courses. The cumulative exposure carried potential toxicity and selection pressure and, plausibly, added a drug-related fever that further obscured the diagnosis.

Procalcitonin elevation has been described in neoplastic fever without infection [33]. Procalcitonin may help identify infection in febrile patients with cancer [34], but its accuracy is only moderate, similar to that of CRP, and it should not be interpreted in isolation [35]; in this patient, the admission value of 0.71 ng/mL was compatible with a bacterial contribution at presentation but did not establish the cause of the fever. The rising ferritin level was likewise non-specific, and the response to an NSAID cannot substitute for tissue diagnosis.

The admission CT offered a biopsy target, but nodal tissue was obtained on HD13 after bone marrow examination; in retrospect, earlier nodal sampling and earlier discontinuation of empirical antimicrobials might have shortened the diagnostic interval and reduced the confounding introduced by antimicrobial therapy. We suggest that, in clinically stable, non-neutropenic patients whose fever persists despite appropriate empirical therapy; whose cultures are negative; and in whom clinical reassessment finds no convincing evidence of an ongoing bacterial infection, clinicians should consider stopping rather than switching or escalating antimicrobials, with close monitoring [4]; should consider drug fever from the antimicrobials already given as well as other non-infectious causes, including occult malignancy [5,6]; and should proceed promptly to imaging and to tissue diagnosis when a target is available. When conventional imaging is unrevealing, ^18^F-FDG PET–CT is the preferred second-line investigation [36,37,38], and comparative and cohort studies support its diagnostic contribution [39,40,41], although in heterogeneous in-patient populations it also prompts many additional examinations [40]. In this patient, CT had already identified a biopsy target, and PET–CT, which was performed after the histological diagnosis, contributed to staging.

This report has limitations. The cause of the fever could not be established: the fever resolved before tumor-directed therapy, so tumor-associated fever could not be confirmed by a therapeutic response, and drug fever remained unconfirmed because defervescence after withdrawal was incomplete and competing causes remained; no rechallenge was performed. The pre-admission fever history relies on the patient’s report; no urine culture obtained before the first course prescribed for the presumed urinary tract infection was available, and extensive antimicrobial exposure may have reduced the sensitivity of cultures. Temperatures between discharge and the start of chemotherapy were not systematically documented, and the absence of fever during chemotherapy should be interpreted cautiously because corticosteroid premedication is routinely given with paclitaxel. The evaluation for tuberculosis was incomplete: mycobacterial culture was performed on a single urine specimen rather than a series of three early-morning specimens, no nucleic acid amplification test was performed on urine or tissue, the lymphadenectomy specimens were not submitted for mycobacterial testing, no interferon-γ release assay was performed, and the thoracic nodes were not sampled. Cytokine profiling, transesophageal echocardiography and venous imaging were not performed, the naproxen challenge was non-standardized, and the extra-abdominal nodal involvement suggested by PET–CT was not histologically confirmed.

## 4. Conclusions

Occult metastatic HGSC may be found in patients with antibiotic-refractory fever and pyelonephritis-like findings, and empirical antimicrobials may add competing causes of fever. In this patient, the cause of the fever remained undetermined: tumor-associated fever, a partially treated urinary tract infection and flomoxef-associated drug fever were all possible, and the diagnosis of malignancy did not by itself explain the fever. When fever persists despite appropriate empirical antimicrobials, cultures are negative and clinical reassessment finds no convincing evidence of an ongoing bacterial infection, clinicians should, in clinically stable, non-neutropenic patients, consider stopping rather than escalating antibiotics with close monitoring, consider drug fever and other non-infectious causes, and proceed promptly to imaging and tissue diagnosis. A transient response to a single, non-standardized naproxen dose cannot be interpreted and should not influence the diagnostic pathway.

## Figures and Tables

**Table 1:** Timeline of the episode of care.

Time	Event
From about 6 months before admission	Intermittent fever; four short courses of oral erythromycin, amoxicillin, doxycycline and clindamycin at other clinics (days −188 to −38)
Day −33 to day −1	Dysuria and right flank pain; outpatient oral antimicrobials for presumed urinary tract infection: cephalexin (days −33 to −30 and −23 to −18), amoxicillin–clavulanate (days −14 to −11), levofloxacin (from day −13) and cephalexin (from day −1)
HD1	Admission; temperature 38.1 °C at triage and 40.1 °C later that day; right costovertebral-angle tenderness; WBC 20,350/µL, CRP 16.6 mg/dL, procalcitonin 0.71 ng/mL; pyuria; CT: para-aortic lymphadenopathy without hydronephrosis
HD2	Intravenous flomoxef started (01:00); temperature 40.0 °C
HD3–HD7	No temperature ≥38.3 °C (maximum 38.2 °C)
HD8	Fever recurred (39.2–40.0 °C)
HD9	00:30, single oral dose of naproxen at 250 mg (40.0 °C 1 h before → 36.5 °C at 2.8 h → 39.1 °C at 7.9 h); 09:00, flomoxef discontinued; bone marrow biopsy (no malignancy)
HD10	High fever continued (up to 39.8 °C)
HD11–HD15	Isolated temperatures of 38.3–39.0 °C (HD11 and HD13–HD15); last temperature ≥38.3 °C on HD15
HD12–HD17	Oral doxycycline for suspected atypical infection (serology negative)
HD13	CT-guided para-aortic lymph-node biopsy
HD16	Histology: metastatic HGSC of the tubo-ovarian type
HD17	PET–CT
HD18	Discharge
Day 30	Repeat urinalysis: no pyuria
Day 41	Primary optimal debulking surgery: no ascites or macroscopic peritoneal disease; metastatic HGSC in 14 of 37 retroperitoneal lymph nodes (surgical–pathological FIGO 2014 stage IIIA1)
Day 56	Readmission with postoperative urinary tract infection
Days 63–187	Six cycles of carboplatin–paclitaxel, with bevacizumab from the third cycle
From day 208	Bevacizumab maintenance until about 8.5 months after diagnosis, followed by surveillance
15 months after diagnosis	No fever documented during chemotherapy or follow-up; CA-125 below 35 U/mL

Days are counted from admission (day 1 = HD1 = day of admission; day −1 = the day before admission). Abbreviations: CA-125, cancer antigen 125; CRP, C-reactive protein; CT, computed tomography; FIGO, International Federation of Gynecology and Obstetrics; HD, hospital day; HGSC, high-grade serous carcinoma; PET–CT, positron emission tomography–computed tomography; WBC, white-cell count.

**Table 2:** Explanations considered for the fever.

Explanation	Findings in Favor	Findings Against	Assessment
Tumor-associated fever	Intermittent fever for about 6 months, preceding all antimicrobial exposure (patient’s report) and persisting through nine courses of six classes; 40.1 °C on admission before intravenous therapy; metastatic HGSC; no persistent infectious focus	Fever subsided by HD16 without tumor-directed therapy, about 1 week after flomoxef withdrawal and during doxycycline treatment (no therapeutic confirmation); pre-admission fever based on the patient’s recall only, without measured temperatures; criteria for neoplastic fever not fulfilled (drug reaction not excluded; naproxen test not performed according to protocol) [10]	Possible; not confirmed
Partially treated urinary tract infection	Dysuria; pyuria with microscopic hematuria; right costovertebral-angle tenderness; leukocytosis, CRP: 16.6 mg/dL and procalcitonin: 0.71 ng/mL on admission; fever and white-cell count fell during the first days of flomoxef treatment; pyuria resolved after antimicrobials were given	Nitrite-negative urine and no abscess or hydronephrosis on CT (neither excludes pyelonephritis); urine culture, obtained during outpatient antimicrobial therapy, yielded only Gram-positive bacilli; right flank findings possibly explained by right para-aortic and intercaval nodal disease; fever recurred during flomoxef treatment; defervescence on HD3–HD7 coincided with the end of oral antimicrobial treatment	Possible contributor at presentation
Drug fever (flomoxef; possibly also the preceding oral antimicrobials)	Recrudescence from the seventh day of flomoxef treatment (HD8); high fever abated within about 36 h of withdrawal; defervescence on HD3–HD7 after the oral prescriptions ended; prior β-lactam exposure (associated with a shorter onset interval for piperacillin but not cefotaxime) [19]	Fever present before any antimicrobial treatment (patient’s report); intermittent fever until HD15 (about 6.5 days after withdrawal) [18,19]; peaks ≥40 °C uncommon and drug fever less frequent in malignancy [19]; no rash, eosinophilia or organ dysfunction (of limited discriminatory value [27])	Possible contributor to the recrudescence (Naranjo score: 4) [28]
Culture-negative endocarditis	Prolonged fever; prior antimicrobial exposure; thickened aortic valve with mild regurgitation	No vegetation on transthoracic echocardiography; fever resolved without antimicrobial therapy directed at endocarditis	Unlikely; not fully excluded (no transesophageal echocardiography)
Atypical or rickettsial infection	Endemic setting; relative bradycardia; fever subsided on the fifth day of doxycycline treatment	Serological tests negative; febrile readings on days 2–4 of doxycycline (HD13–HD15)	Unlikely
Tuberculosis (nodal or genitourinary)	Endemic setting; prolonged fever; pyuria with microscopic hematuria and a non-diagnostic culture; lymphadenopathy; FDG-avid mediastinal and hilar nodes; prior levofloxacin (potential confounder)	Nodal histology of metastatic HGSC; no granulomas described; acid-fast stain and mycobacterial culture of the nodal biopsy negative; sputum (×3) and single urine mycobacterial cultures negative; thoracic nodes regressed after three cycles of chemotherapy without antituberculous therapy	Unlikely; not excluded (single urine culture rather than three early-morning specimens; no NAAT or IGRA; lymphadenectomy specimens not tested; thoracic nodes not sampled)
Lymphoma or hemophagocytic lymphohistiocytosis	Fever; lymphadenopathy; rising ferritin and LDH levels	Nodal immunophenotype of HGSC; bone marrow without malignancy (EBER negative); no cytopenia meeting the HLH criteria	Very unlikely
Other healthcare-associated causes (catheter-related infection, *Clostridioides difficile* infection, pneumonia, venous thromboembolism, and procedure-related fever)	New fever during hospitalization; elevated D-dimer levels (HD3); one febrile episode on the day of the nodal biopsy	Normal catheter site; no diarrhea; no pneumonia on chest imaging; blood cultures negative	Unlikely as the main cause; venous thromboembolism not excluded
Connective-tissue disease	ANA 1:320 (fine speckled); anti-SSA/Ro52 and antimitochondrial antibodies positive	No clinical features on rheumatological assessment; fever resolved without immunosuppressive therapy	Unlikely

Abbreviations: ANA, antinuclear antibody; CRP, C-reactive protein; CT, computed tomography; EBER, Epstein–Barr virus-encoded RNA; FDG, ^18^F-fluorodeoxyglucose; HD, hospital day; HGSC, high-grade serous carcinoma; HLH, hemophagocytic lymphohistiocytosis; IGRA, interferon-γ release assay; LDH, lactate dehydrogenase; NAAT, nucleic acid amplification test.

**Table 3:** Summary of key studies on the naproxen test in the differential diagnosis of fever.

Study (Year)	Design and Population	Main Result	Interpretation
Chang & Gross (1984) [16]	Case series; *n* = 22 hospitalized patients with cancer (20) or connective-tissue disease (2) and fever of undetermined origin, >7 days in all but one [10,16]	A total of 14/15 with neoplastic fever showed prompt, complete and sustained lysis within 24 h, 0/5 with infection responded, and the 2 with connective-tissue disease showed partial lysis.	Origin of the “naproxen test” concept; continued dosing (250 mg twice daily), with complete response defined as defervescence within 24 h and normal temperature for >5 days while on naproxen [16]
Vanderschueren et al. (2003) [17]	Retrospective single-center study; *n* = 77 with prolonged unexplained fever (not cancer-restricted); naproxen or another NSAID for ≥2 days at physician-chosen doses	Neoplastic fever in 11/77 (14%); defervescence in 6/11 (55%) with neoplastic vs. 38% with non-neoplastic fever (no significant difference) [10,17]	Challenges validity of the test in undifferentiated FUO
Zhang et al. (2019) [30]	Systematic review and meta-analysis; 15 studies, *n* = 582; fever in patients with cancer	Pooled proportion with successful treatment: 94.1% in neoplastic fever, 79.8% in suspected neoplastic fever (450 patients) and 67.7% in FUO (227 patients) from different study subsets	Treatment-response data, which were mostly observational, from patients with cancer; not diagnostic accuracy

Abbreviations: FUO, fever of unknown origin; NSAID, non-steroidal anti-inflammatory drug.

## Data Availability

The original contributions presented in this study are included in the article. Further inquiries regarding the de-identified clinical data can be directed to the corresponding author. The de-identified temperature and laboratory data underlying Figure 1 and the code used to plot them are available from the corresponding author upon request.

## Abbreviations

The following abbreviations are used in this manuscript:

CA-125	Cancer antigen 125
CRP	C-reactive protein
CT	Computed tomography
FIGO	International Federation of Gynecology and Obstetrics
FUO	Fever of unknown origin
HD	Hospital day
HGSC	High-grade serous carcinoma
IL	Interleukin
NSAID	Non-steroidal anti-inflammatory drug
PET–CT	Positron emission tomography–computed tomography
TNF	Tumor necrosis factor
